# Supplementary material for: Deletion of wfs1 Impairs Oligodendrocyte Precursor Cells Dorsal Distribution and Myelination Through the wfs1-hmgcs1 Axis in Zebrafish
Source: Biology (Basel). 2026 Aug 16;15(16):1402. doi: 10.3390/biology15161402 (PMC13510151; doi:10.3390/biology15161402)
Supplement: Supplementary file 1 [file biology-15-01402-s001.zip › File S1-Transcriptome sequencing data and differentially expressed genes (DEGs) in wfs1-- zebrafish at 5 dpf/2.KEGG/wfs1KOvsWT/UP/src/dre00280.html]

dre00280


- dre:797311

- Up regulated genes

797311(1.5489285772488)

- dre:777632

- Up regulated genes

777632(1.55582226921231)

- dre:437019

- Up regulated genes

437019\_1(2.62419743690105)

- dre:368239

- Up regulated genes

368239(1.14034759711527)

- dre:394130

- Up regulated genes

394130\_1(2.23229468222569)

Close
